# Tripled and sustained change in pregnancy-related annual mortality rates with systemic lupus erythematosus involvement: a nationwide temporal trends study, Brazil, 2006–2022

**DOI:** 10.1186/s42358-025-00445-8

**Published:** 2025-03-14

**Authors:** Rodrigo Poubel Vieira de Rezende, Bruno Santos Caxias, Anna Beatriz da Silva Rodrigues, Omar Hazem Ashmawi, Luiz Eduardo da Costa Oliveira

**Affiliations:** 1 Department of Clinical Medicine, Universidade Federal Fluminense, Rio de Janeiro, Brazil; 2 Faculdade de Medicina, Universidade Federal Fluminense, Rua Desembargador Athayde Parreiras, 100, Bairro de Fátima, Niterói, Rio de Janeiro, CEP 24070-090 Brazil

**Keywords:** Systemic lupus erythematosus, Epidemiologic trends, Pregnancy complications, Puerperium, Reproductive-age women, Maternal death

Significant progress has been made in recent decades in improving the survival and quality of life of patients with systemic lupus erythematosus (SLE) [1]. However, data on temporal trends in pregnancy-related mortality among women with SLE remain scarce [2], particularly in Brazil, where no such studies have been conducted. This ecological study aimed to assess the impact of SLE on mortality among women of childbearing age in Brazil due to pregnancy, childbirth, and puerperium (PCP)-related complications.

Mortality data for Brazil, covering the period from 2006 to 2022, were extracted from the Mortality Information System, linked to the Brazilian Ministry of Health (publicly available at https://datasus.saude.gov.br). For trend analysis, data on resident women aged 10–49 years were obtained from the same Brazilian government website and the United Nations Population Division. The 2014 world female population aged 10–49 years was selected as the standard for age adjustment, based on the 2022 revision (publicly available at https://www.un.org/development/desa/pd).

During this period, 31,867 deaths were identified among women aged 10 to 49 years, in which the underlying cause of death (UCD) was attributed to PCP-related complications (Chapter XV codes O00-O99, according to the 10th revision of the International Classification of Diseases [ICD-10]). Of these, the ICD-10 code for SLE (M32) was reported as a diagnostic mention or non-underlying cause in 273 cases (0.9%), hereafter referred to as “SLE-related cases” (see Supplementary spreadsheet).

Table 1 compares UCD distributions between all women of childbearing age and SLE-related cases based on pre-specified ICD-10 Chapter XV groupings. SLE-related cases were less frequent in all groupings except for “Other obstetric conditions, not elsewhere classified” (O94-O99), which includes the codes for late maternal deaths (O96-O97). Regarding late puerperium deaths, 68 cases (24.9%) were SLE-related versus 2,685 (8.4%) among all cases (*p* < 0.0001, Fisher’s exact test).

**Table 1:** Comparison of underlying cause of death by pre-specified ICD-10 chapter XV groupings (O00-O99: pregnancy, childbirth, and the puerperium) between SLE-related and total cases in women aged 10–49 years, Brazil, 2006–2022

Chapter XV groupings	ICD-10 code	Total cases *N* = 31,867	SLE cases *N* = 273	*P*-value ^a^
Pregnancy with abortive outcome	O00-O08	2,217 (7.0)	5 (1.8)	0.0003
Edema, proteinuria and hypertensive disorders in pregnancy, childbirth and the puerperium	O10-O16	6,071 (19.1)	29 (10.6)	0.0002
Other maternal disorders predominantly related to pregnancy	O20-O29	1,191 (3.7)	2 (0.7)	0.003
Maternal care related to the fetus and amniotic cavity and possible delivery problems	O30-O48	1,746 (5.5)	2 (0.7)	< 0.0001
Complications of labor and delivery	O60-O75	4,391 (13.8)	6 (2.2)	< 0.0001
Delivery	O80-O84	0	0	NA
Complications predominantly related to the puerperium	O85-O92	3,820 (12.0)	18 (6.6)	0.0047
Other obstetric conditions, not elsewhere classified	O94-O99	12,431 (39.0)	211 (77.3)	< 0.0001

Results are shown as absolute number (percentage)

^a^ P-values were calculated using Fisher`s exact test

ICD-10: International Classification of Diseases, tenth revision; NA: not applicable

For the temporal trend analysis (2006–2022), the joinpoint method calculated the annual percentage change (APC) in the age-adjusted mortality rate (per 100,000) of women aged 10–49 years due to PCP-related complications [3]. The APC was 2% (95% confidence interval [CI]: 0 to 4.0%) for all cases versus 5.6% (95% CI: 2.0–9.4%) for SLE-related cases (see supplementary files for detailed results of the APC calculation). No joinpoints, or significant inflection points where the trend changes direction, were identified (see Fig. 1).

**Fig. 1:**
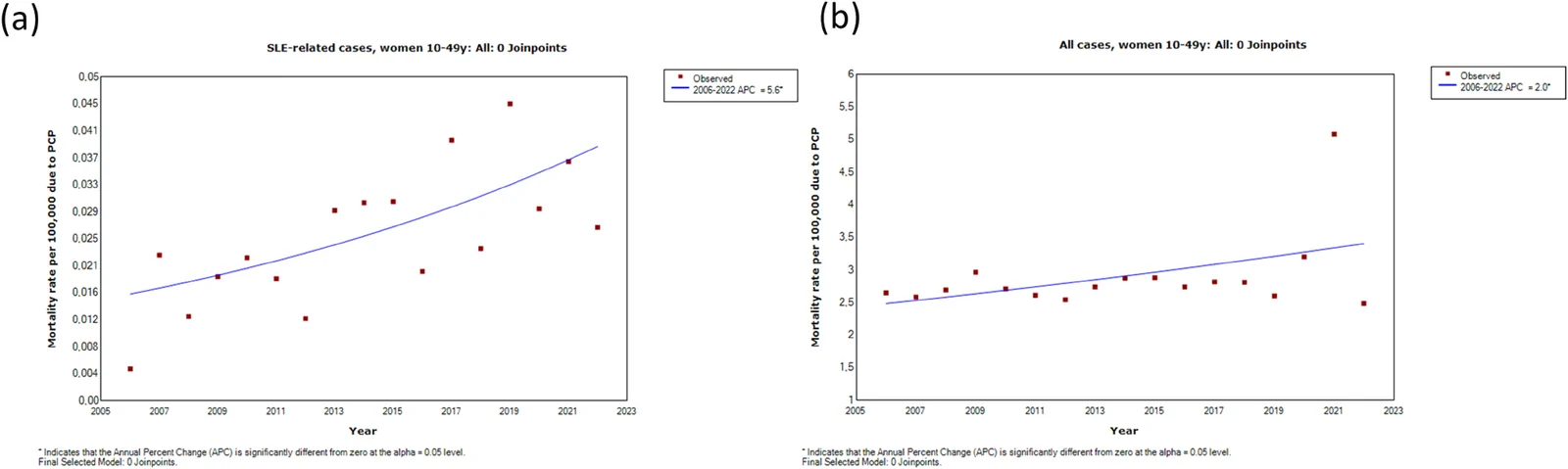
Age-adjusted mortality rates per 100,000 due to pregnancy, childbirth, and puerperium (PCP)-related complications in: (**a**) systemic lupus erythematosus (SLE)-related cases and (**b**) all cases among women aged 10–49 years in the Brazilian general population, 2006–2022

This study has limitations. As our analyses rely on secondary data, we cannot rule out the possibility of misclassification or underreporting in mortality records, which may affect the true burden of SLE-related pregnancy mortality. Nevertheless, as the Mortality Information System is the official national database, it remains a valuable resource for population-based analyses.

In conclusion, this study demonstrated that (1) the APC for pregnancy-related deaths among women of childbearing age was nearly tripled with SLE involvement, and (2) late puerperium deaths were disproportionately higher among SLE-related cases. These findings highlight the need for tailored care strategies for pregnant women with SLE in Brazil, with particular focus on the late puerperium. Given the study’s ecological design, our results should be interpreted with caution and validated in prospective cohort studies.

## Electronic supplementary material

Below is the link to the electronic supplementary material.

Supplementary Material 1

Supplementary Material 2

Supplementary Material 3

## Data Availability

All data generated or analyzed during this study are included in this published article and its supplementary information files.
