# Supplementary material for: Tripled and sustained change in pregnancy-related annual mortality rates with systemic lupus erythematosus involvement: a nationwide temporal trends study, Brazil, 2006–2022
Source: Adv Rheumatol. 2025 Mar 14;65:12. doi: 10.1186/s42358-025-00445-8 (PMC13488728; doi:10.1186/s42358-025-00445-8)
Supplement: Supplementary file 1 [file 42358_2025_445_MOESM1_ESM.pdf]

Number of Joinpoints: 0 (Final Selected Model)

Model Statistics							
**Cohort**	**Number of Joinpoints**	**Number of Observations**	**Number of Parameters**	**Degrees of Freedom**	**Sum of Squared Errors**	**Mean Squared Error**	**Autocorrelation Parameter**
	0	17	2	15	949.69262	63.31284	Uncorrelated

Estimated Regression Coefficients (Beta)

Standard Parameterization					
**Cohort**	**Parameter**	**Param Estimate**	**Standard Error**	**Test Statistic (t)**	**Prob > |t|**
	Intercept 1	-38.618385	18.182089	-2.123980	0.050711
	Slope 1	0.019705	0.009026	2.183197	0.045324

- The statistic could not be calculated.

General Parameterization					
**Cohort**	**Parameter**	**Param Estimate**	**Standard Error**	**Test Statistic (t)**	**Prob > |t|**
	Intercept 1	-38.618385	18.182089	-2.123980	0.050711
	Slope 1	0.019705	0.009026	2.183197	0.045324

- The statistic could not be calculated.

Annual Percent Change (APC)								
**Cohort**	**Segment**	**Lower EndPoint**	**Upper Endpoint**	**APC**	**Lower CI**	**Upper CI**	**Test Statistic (t)**	**Prob > |t|**
	1	2006	2022	2.0*	0.0	4.0	2.2	0.045

* Indicates that the Annual Percent Change (APC) is significantly different from zero at the alpha = 0.05 level.

- The statistic could not be calculated.

, Number of Joinpoints: 0 (Final Selected Model) continued...

Average Annual Percent Change (AAPC)								
**Cohort**	**Range**	**Lower EndPoint**	**Upper Endpoint**	**AAPC**	**Lower CI**	**Upper CI**	**Test Statistic ***	**P-Value ***
	Full Range	2006	2022	2.0*	0.0	4.0	2.2	< 0.1

* Indicates that the AAPC is significantly different from zero at the alpha = 0.05 level.
~ If the AAPC is within one segment, the t-distribution is used. Otherwise, the normal (z) distribution is used.

- The statistic could not be calculated.

Model Selection Method	
**Cohort**	**Model Selection Method**
	Permutation Test

Test for Number of Joinpoints								
**Cohort**	**Test Number**	**Null Hypothesis**	**Alternate Hypothesis**	**Number Degrees of Freedom**	**Denominator Degrees of Freedom**	**Number of Permutations**	**P-Value**	**Significance Level ~**
	#1	0 Joinpoint(s) ^	3 Joinpoint(s)	6	9	4500	0.9477778	0.0166667
	#2	0 Joinpoint(s) ^	2 Joinpoint(s)	4	11	4500	0.8946667	0.0166667
	#3	0 Joinpoint(s) ^	1 Joinpoint(s)	2	13	4500	0.4971111	0.0166667
Final Selected Model: 0 Joinpoint(s)								

^ Final Selected Model

~ Significance level for individual test
