# Supplementary material for: Tripled and sustained change in pregnancy-related annual mortality rates with systemic lupus erythematosus involvement: a nationwide temporal trends study, Brazil, 2006–2022
Source: Adv Rheumatol. 2025 Mar 14;65:12. doi: 10.1186/s42358-025-00445-8 (PMC13488728; doi:10.1186/s42358-025-00445-8)
Supplement: Supplementary file 2 [file 42358_2025_445_MOESM2_ESM.pdf]

Number of Joinpoints: 0 (Final Selected Model)

Model Statistics							
**Cohort**	**Number of Joinpoints**	**Number of Observations**	**Number of Parameters**	**Degrees of Freedom**	**Sum of Squared Errors**	**Mean Squared Error**	**Autocorrelation Parameter**
	0	17	2	15	22.55875	1.50392	Uncorrelated

Estimated Regression Coefficients (Beta)					
**Standard Parameterization**					
**Cohort**	**Parameter**	**Param Estimate**	**Standard Error**	**Test Statistic (t)**	**Prob > |t|**
	Intercept 1	-113.690273	33.350557	-3.408947	0.003886
	Slope 1	0.054621	0.016548	3.300844	0.004851

- The statistic could not be calculated.

General Parameterization					
**Cohort**	**Parameter**	**Param Estimate**	**Standard Error**	**Test Statistic (t)**	**Prob > |t|**
	Intercept 1	-113.690273	33.350557	-3.408947	0.003886
	Slope 1	0.054621	0.016548	3.300844	0.004851

- The statistic could not be calculated.

Annual Percent Change (APC)								
**Cohort**	**Segment**	**Lower EndPoint**	**Upper Endpoint**	**APC**	**Lower CI**	**Upper CI**	**Test Statistic (t)**	**Prob > |t|**
	1	2006	2022	5.6*	2.0	9.4	3.3	0.005

* Indicates that the Annual Percent Change (APC) is significantly different from zero at the alpha = 0.05 level.

- The statistic could not be calculated.

, Number of Joinpoints: 0 (Final Selected Model) continued...

Average Annual Percent Change (AAPC)								
**Cohort**	**Range**	**Lower EndPoint**	**Upper Endpoint**	**AAPC**	**Lower CI**	**Upper CI**	**Test Statistic ***	**P-Value ***
	Full Range	2006	2022	5.6*	2.0	9.4	3.3	< 0.1

* Indicates that the AAPC is significantly different from zero at the alpha = 0.05 level.
~ If the AAPC is within one segment, the t-distribution is used. Otherwise, the normal (z) distribution is used.

- The statistic could not be calculated.

Model Selection Method	
**Cohort**	**Model Selection Method**
	Permutation Test

Test for Number of Joinpoints								
**Cohort**	**Test Number**	**Null Hypothesis**	**Alternate Hypothesis**	**Number Degrees of Freedom**	**Denominator Degrees of Freedom**	**Number of Permutations**	**P-Value**	**Significance Level ~**
	#1	0 Joinpoint(s) ^	3 Joinpoint(s)	6	9	4500	0.8286667	0.0166667
	#2	0 Joinpoint(s) ^	2 Joinpoint(s)	4	11	4500	0.7148889	0.0166667
	#3	0 Joinpoint(s) ^	1 Joinpoint(s)	2	13	4500	0.3180000	0.0166667
Final Selected Model: 0 Joinpoint(s)								

^ Final Selected Model

~ Significance level for individual test
