# Supplementary material for: Tripled and sustained change in pregnancy-related annual mortality rates with systemic lupus erythematosus involvement: a nationwide temporal trends study, Brazil, 2006–2022
Source: Adv Rheumatol. 2025 Mar 14;65:12. doi: 10.1186/s42358-025-00445-8 (PMC13488728; doi:10.1186/s42358-025-00445-8)
Supplement: Supplementary file 3 [file 42358_2025_445_MOESM3_ESM.pdf]

Year	Age group	death count: SLE-related cases (Brazil, women, 10-49y)	death count: all cases (Brazil, women, 10-49y)	female population - Brazil	2014 world female population (standard)
2006	10-14a	0	17	8514991	298687000
2006	15-19a	0	250	8749890	290774000
2006	20-29a	3	685	17199862	588879000
2006	30-39a	0	554	14228729	510706000
2006	40-49a	0	124	12046496	463624000
2007	10-14a	0	12	8557380	298687000
2007	15-19a	1	218	8661956	290774000
2007	20-29a	10	670	17356992	588879000
2007	30-39a	3	567	14473291	510706000
2007	40-49a	0	135	12320193	463624000
2008	10-14a	0	11	8599184	298687000
2008	15-19a	0	244	8591310	290774000
2008	20-29a	4	697	17454089	588879000
2008	30-39a	3	573	14743287	510706000
2008	40-49a	1	160	12572815	463624000
2009	10-14a	0	17	8605252	298687000
2009	15-19a	0	254	8550293	290774000
2009	20-29a	7	815	17496816	588879000
2009	30-39a	4	638	15037265	510706000
2009	40-49a	1	151	12800599	463624000
2010	10-14a	0	25	8554717	298687000
2010	15-19a	0	246	8545250	290774000
2010	20-29a	10	738	17490167	588879000
2010	30-39a	4	585	15352243	510706000
2010	40-49a	0	132	13002615	463624000
2011	10-14a	0	16	8455975	298687000
2011	15-19a	2	236	8545134	290774000
2011	20-29a	6	687	17479630	588879000
2011	30-39a	4	608	15652960	510706000
2011	40-49a	0	131	13183752	463624000
2012	10-14a	0	25	8283444	298687000
2012	15-19a	2	216	8570279	290774000
2012	20-29a	5	677	17429174	588879000
2012	30-39a	1	584	15969636	510706000
2012	40-49a	0	143	13343647	463624000
2013	10-14a	0	14	8089460	298687000
2013	15-19a	4	240	8598599	290774000
2013	20-29a	8	688	17355051	588879000
2013	30-39a	7	698	16284046	510706000
2013	40-49a	0	147	13495654	463624000
2014	10-14a	0	20	7907189	298687000
2014	15-19a	1	235	8595020	290774000
2014	20-29a	10	744	17281589	588879000
2014	30-39a	9	765	16569509	510706000
2014	40-49a	0	124	13659760	463624000
2015	10-14a	0	14	7760725	298687000
2015	15-19a	4	239	8538650	290774000
2015	20-29a	8	775	17223743	588879000
2015	30-39a	7	726	16808569	510706000
2015	40-49a	1	142	13848875	463624000
2016	10-14a	0	16	7628594	298687000
2016	15-19a	0	211	8440520	290774000
2016	20-29a	8	723	17180164	588879000
2016	30-39a	5	703	17002486	510706000
2016	40-49a	0	156	14060069	463624000
2017	10-14a	0	14	7522151	298687000
2017	15-19a	3	208	8268820	290774000
2017	20-29a	15	702	17154918	588879000
2017	30-39a	8	791	17150677	510706000
2017	40-49a	0	157	14292029	463624000
2018	10-14a	0	13	7430076	298687000
2018	15-19a	2	236	8075784	290774000
2018	20-29a	9	690	17134569	588879000
2018	30-39a	4	750	17255161	510706000
2018	40-49a	0	173	14544866	463624000
2019	10-14a	0	14	7343636	298687000
2019	15-19a	1	199	7894402	290774000
2019	20-29a	23	642	17095158	588879000
2019	30-39a	4	716	17320599	510706000
2019	40-49a	1	155	14816664	463624000
2020	10-14a	0	8	7241977	298687000
2020	15-19a	2	172	7748661	290774000
2020	20-29a	11	767	17021793	588879000
2020	30-39a	6	1000	17351596	510706000
2020	40-49a	0	199	15104223	463624000
2021	10-14a	0	13	7162974	298687000
2021	15-19a	2	252	7617127	290774000
2021	20-29a	12	1243	16925725	588879000
2021	30-39a	10	1605	17346100	510706000
2021	40-49a	0	289	15406326	463624000
2022	10-14a	0	12	7126965	298687000
2022	15-19a	2	139	7511038	290774000
2022	20-29a	9	662	16780577	588879000
2022	30-39a	6	662	17300539	510706000
2022	40-49a	0	163	15723687	463624000
